# Suppression of the increasing level of acetylcholine-stimulated intracellular Ca^2+^ in guinea pig airway smooth muscle cells by mabuterol

**DOI:** 10.3892/br.2015.502

**Published:** 2015-08-04

**Authors:** XIRUI SONG, CHAO ZHAO, CAILING DAI, YANXIN REN, NAN AN, HUIMIN WEN, LI PAN, MAOSHENG CHENG, YUYANG ZHANG

**Affiliations:** 1 Department of Pharmacology, School of Life Science and Biopharmaceutics, Shenyang Pharmaceutical University, Shenyang, Liaoning 110016, P.R. China; 2 Department of Medicine Chemistry, School of Pharmaceutical Engineering, Shenyang Pharmaceutical University, Shenyang, Liaoning 110016, P.R. China; 3 Key Laboratory of Structure Based Drug Design and Discovery, Ministry of Education, Shenyang, Liaoning 110016, P.R. China

**Keywords:** guinea pigs, airway smooth muscle cells, mabuterol, intracellular Ca^2+^, acetylcholine

## Abstract

The present study aimed to establish an effective method for the *in vitro* culture of guinea pig airway smooth muscle (ASM) cells, and also investigate the suppressive effect of mabuterol hydrochloride (Mab) on the increased level of intracellular Ca^2+^ in ASM cells induced with acetylcholine (Ach). Two different methods, i.e. with or without collagenase to pretreat tracheal tissues, were applied to the manufacture of ASM cells. Cell viability was determined with the 3-(4,5-dimethylthinazol-2-yl)-2,5-diphenyltetrazolium bromide assay. Immunocytochemistry and immunofluorescence were used for the identification of ASM cells. Different concentration levels (10^−3^, 10^−4^, 10^−5^, 10^−6^ and 10^−7^ mmol/l) of Mab were administered 5 min before Ach (10^−4^ M) treatment, respectively. The Ca^2+^ fluorescent probe, Fura-2/AM or Fluo-3/AM were applied to the inspection of Ca^2+^ fluorescent intensity with Varioskan Flash, immunocytometry systems and an inverted system microscope, respectively. The results showed that the fresh method, in which isolated tracheal tissues were previously treated with collagenase for 20 min, was more advantageous for the preparation of guinea pig ASM cells compared to when the enzyme was not used. The time for the ASM cells to initially migrate out of the ‘tissue blocks’ and the culture having to be generated due to the thick cell density was significantly less. On identification with immunocytochemistry or immunofluorescent staining, >95% of the cells were ASM cells. Mab (10^−3^−10^−7^ mmol/l) significantly suppressed the elevation of intracellular Ca^2+^ induced by Ach in a concentration-dependent manner. The inhibitory rates of intracellular Ca^2+^ by different concentrations of Mab, from low to high, were 14.93, 24.73, 40.06, 48.54 and 57.13%, respectively, when Varioskan Flash was used for determination. In conclusion, this novel method has a shorter harvesting period for ASM cells. Mab can suppress the increasing level of intracellular Ca^2+^ induced by Ach in guinea pig ASM cells. Further investigation into the precise mechanisms of action is required.

## Introduction

Asthma is one of the most common chronic diseases characterized by airway hyperresponsiveness (AHR) and airway remodeling. It has been well established that airway smooth muscle (ASM) cells are the main components of the respiratory tract. They are believed to have a role in the pathogenesis of asthma through their contractile properties (1). Additionally, it is widely accepted that the cells act as immunomodulation, which contribute to the inflammation of the respiratory airway and structural alterations via inflammatory and immunological factors associated with asthma (2). Noble *et al* (3) proposed that ASM contraction, in combination with cellular mechanotransduction and novel contraction-inflammation synergies, contributed to the heterogeneous pathogenesis of asthma. The contraction is the basis of ASM function. It is well known that ASM contraction is regulated by secondary messengers, such as guanosine 3′,5′-cyclic phosphate, cyclic adenosine monophosphate and Ca^2+^ (4). Among them, Ca^2+^ is an important secondary messenger that regulates miscellaneous responses in ASM cells, such as contraction, relaxation, proliferation, migration and cytokine secretion. Elevation of the Ca^2+^ level is derived from intracellular Ca^2+^ release out of the sarcoplasmic reticulum (SR) and extracellular Ca^2+^ influx (5,6). Wang *et al* (7) identified that the change of cytosolic Ca^2+^ level determined the primary-signal-regulating contractile function of ASM cells. It is clear that Ca^2+^ is a key factor for assessing the efficacy of drugs used in asthma.

**Figure 1.**
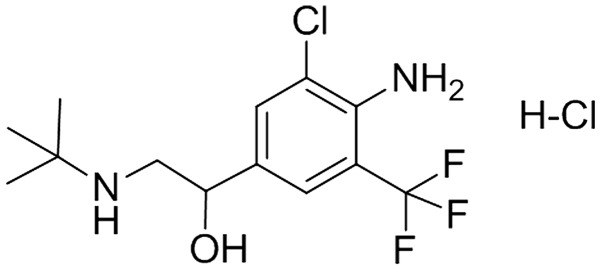
Molecular structure of mabuterol hydrochloride.

Mabuterol hydrochloride (Mab) (Fig. 1) as a novel β_2_-agonist with high selectivity has good pharmacokinetic properties, such as an orally complete absorption and a long duration of action, and it has been clinically used as a bronchodilator in the treatment of asthma (8). Pharmacodynamic studies of Mab have been conducted since it was first synthesized by German scholars in 1984. Osada *et al* (9) studied the effect of Mab on the cardiovascular system and smooth muscle organs of rats, cats and dogs and made a comparison with those of isoprenaline, salbutamol and procaterol. They found that the drug did not influence α-adrenergic, acetylcholine (Ach) and histamine receptors, and was a specific β_2_ blocker with no β_1_-stimulation. The effect on blood pressure and peripheral vascular resistance in dogs was 365 and 118 times less compared to isoprenaline. Additionally, it was shown in the study by Akahane *et al* (10) that Mab, when injected into the sinus node artery of the isolated atrium, dose-dependently increased the atrial rate and contractile force, which were inhibited by a selective β_2_-receptor antagonist, ICI 118551, and only slightly attenuated by atenolol. These weak-positive chronotropic and inotropic effects were clearly produced by stimulating β_2_-adrenoceptors on the perfused canine right atrium. However, there is limited literature regarding the precise mechanism of action for Mab.

In the present study, a renewed and stable method of culturing guinea pig ASM cells was established. The suppression of increasing intracellular calcium by Mab was investigated with several detection methods and two agents Fura-2/AM, as well as Fluo-3/AM as a Ca^2+^ indicator.

## Materials and methods

### 

#### Animals

Male or female Hartley guinea pigs, weighing 150–200 g, were provided by the Experimental Animal Center of Shenyang Pharmaceutical University (Shenyang, Liaoning, China). Animals were bred in a facility controlled by temperature (26±3°C), relative humidity (50±5%) and light (14 and 10 h of light and dark), with free access to food and water, with added vitamin C. All the experimental procedures in the present study were carried out in accordance with the Internationally Accepted Principles and the Guidelines for the Care and Use of Animal Center of Shenyang Pharmaceutical University.

#### Drugs and chemicals

Mab was supplied by the Pharmaceutical Engineering Department, Shenyang Pharmaceutical University (enantiomeric excess >99%). Ach was purchased from Sinopharm Chemical Reagent Co., Ltd. (Shanghai, China). Dulbecco's modified Eagle's medium (DMEM) and Hanks' balanced salt solution (HBSS) were purchased from Gibco-BRL (Carlsbad, CA, USA) and type I collagenase from Beijing Solarbio Science and Technology Co., Ltd. (Beijing, China). Fetal bovine serum (FBS) was produced by Tianjin Hualida Biotechnology Co., Ltd. (Tianjin, China). Triton X-100 and 3-(4,5-dimethylthinazol-2-yl)-2,5-diphenyltetrazolium bromide (MTT) were obtained from Amresco LLC (Solon, OH, USA). Mouse anti-α-smooth muscle actin (α-SMA), 5% bovine serum albumin (BSA), streptavidin-biotin complex (SABC) immunohistochemical staining kit and 3,3′ diaminobenzidine (DAB) chromogenic reagent kit were all purchased from Wuhan Boster Biological Technology Co., Ltd. (Wuhan, China). Fura-2/AM, Fluo-3/AM and fluorescein isothiocyanate (FITC)-labeled goat anti-mouse immunoglobulin G (IgG) were from Beyotime Institute of Biotechnology (Haimen, Jiangsu, China).

#### Applying two different methods to the culture

One of the methods used collagenase to pretreat tracheal tissues (CPTT) and the other did not. Freshly dispersed tracheal smooth muscle strips of guinea pig were prepared as described previously (11). Briefly, tracheal samples of several guinea pigs were mechanically isolated and instantly placed in 4°C HBSS. They were dissected free of adhering fat and other connective tissues. Smooth muscle strips without any tracheal cartilage were obtained and cut into 1-mm^3^ pieces. The ASM pieces were randomly divided into two groups. Half of the pieces were moved into one culture flask with a cell growth area of 25 cm^2^ and put evenly on the inside wall. The culture process followed the steps of the traditional method. The other half were placed into another flask after digestion with 2% type I collagenase for 20 min (37°C, 5% CO_2_) and the culture process followed the steps of the CPTT method. A total of 2 ml of DMEM containing 20% FBS, 100 IU/ml penicillin, 100 IU/ml streptomycin and 2 mmol/l L-glutamine was added to immerse the ASM pieces when the edge of ASM had dried and began to tightly attach to the surface of the flask. All the flasks were placed in the humidified atmosphere containing 5% CO_2_ at 37°C and the medium was partially replaced every 3 days in accordance with the routine procedure in cell culture. Cell growth was observed daily. The time for the cells to initially migrate out of the ASM pieces as well as the culture having to be generated due to the thick density of the cells in the flask was recorded. The pieces, out of which ASM cells first migrate, were moved into a new flask when enough cells were harvested. They could be repetitively used in the production of the ASM cells ≤3 times.

#### Cell viability assay

When the cell density was ~80%, the ASM pieces were moved into another culture flask to be fully prepared as described above. Subsequently, the cells were detached with the mixed solution of 0.25% trypsin and 0.02% EDTA at 37°C for 3 min in preparation for cell generation. The MTT assay was used to determine cell viability (12). Briefly, 500 µl of the third generation of cell suspension at a density of 1×10^4^ cells/ml was seeded in 96-well plates and incubated at 37°C with 5% CO_2_ for 1, 2, 3, 4, 5, 6 and 7 days, respectively. When the MTT assay was conducted, 150 µl of phosphate-buffered saline (PBS) with 0.5 mg/ml MTT was added to the medium in each well and incubated at 37°C with 5% CO_2_ for 4 h. Subsequently, the supernatant was removed by aspiration and dimethyl sulfoxide was added into each well. The reaction was sustained for 10 min at room temperature. The amount of MTT formazan was quantified by measuring optical density (OD) at 492 nm with a Varioskan Flash (Thermo Fisher Scientific, Inc., Rockford, IL, USA). A cell growth curve was generated with GraphPad Prism 5 (GraphPad Software, San Diego, CA, USA).

#### Identification of guinea pig ASM cells

To confirm that the cells were ASM cells and not epithelial cells or fibroblasts, homogeneity was confirmed with α-SMA according to a previously described method (13). Briefly, ASM cells of generation three, four or five were cultivated in a 24-well plate at a density of 10,000 cells/well. They were rinsed with 0.01 M PBS (pH 7.2–7.4), fixed with 4% phosphate-buffered paraformaldehyde for 30 min, and attached to the bottom of the plate and grew in a good condition. Subsequently, 5% BSA was added to block the non-specific proteins for 20 min after they were treated with 0.25% Triton X-100 for 10 min at room temperature. The cells were incubated with α-SMA (1:200) in a wet box at 4°C overnight. Following this, they were rinsed 3 times with 0.01 M PBS. The plate used for immunocytochemistry was incubated with goat anti-mouse IgG (1:200) for 20 min, treated with SABC for 30 min at 37°C and the chromogenic reaction was conducted with DAB for 8 min. The image was observed under an inverted system microscope (IX71; Olympus, Tokyo, Japan). The plate used for immunofluorescence was incubated with FITC-labeled goat anti-mouse IgG for 60 min at 37°C. The immunofluorescent image was observed under the inverted microscope with an absorption peak at 492 nm and emission peak at 520 nm, and the data were saved.

#### Determination of intracellular Ca^2+^

##### Measurement with Fura-2/AM

Intracellular Ca^2+^ was indicated with a fluorescent molecular probe, Fura-2/AM, as described previously (14). In brief, the cells were carefully moved into a sterile eppendorf tube at a density of ~2×10^5^ cells/tube. Subsequently, they were preloaded with Fura-2/AM for 60 min in a humidified incubator (37°C, 5% CO_2_) at a final concentration of 5 µmol/l in DMEM (pH 7.2–7.4) supplemented with 10% FBS. The cells loaded with Fura-2/AM were rinsed with 0.2% BSA twice. The eppendorf tube was centrifuged at 220 × g for 5 min at room temperature and HBSS without Ca^2+^ was used to suspend the cell pellets. One section of the cells was loaded with Fura-2/AM in 100 µl of suspension and was moved into black 96-well culture plates (Corning Life Sciences, Tewksbury MA, USA) at the density of ~2×10^4^ cells/well for the determination of Ca^2+^ fluorescence intensity (F) with the Varioskan Flash under the condition of an excitation wavelength at 340 nm and emission wavelength at 510 nm. The inhibitory rate of calcium was calculated according to the equation: Calcium inhibitory rate (%) = (F_340 control_ − F_340 mabuterol_)/F_340control_ × 100%. A second section of the cells was loaded with Fura-2/AM in 200 µl of suspension and was transferred into 24-well culture plates to obtain Ca^2+^ fluorescent images at the ultraviolet region under an inverted system microscope (IX71; Olympus).

#### Measurement with Fluo-3/AM

A total of 2 µmol/l of the Ca^2+^-sensitive Fluo-3/AM was required according to the manufacturer's instruction when the intracellular Ca^2+^ level was determined with flow cytometry. Cells at the density of 3×10^6^/ml were incubated at 37°C for 45 min in the dark after the treatment with Mab plus Ach, as described previously. The cells were gently rinsed with HBSS without Ca^2+^ 3 times. When Fluo-3/AM binds to cytoplasmic-free calcium, the complex emits green fluorescence under the stimulation of the 488 nm line of an argon ion laser. The fluorescent intensity at 525 nm was determined at 37°C with the Becton-Dickinson Immunocytometry system (FACSCalibur; BD Biosciences, San Jose, CA, USA) and the light signal was converted into an electric signal with linear amplification.

#### Statistical analysis

Results are expressed as mean ± standard error of the mean and statistical comparisons among groups were performed with one-way analysis of variance followed by least significant difference or independent samples t-test using SPSS 16.0 (SPSS Inc., Chicago, IL, USA). P<0.05 was considered to indicate a statistically significant difference in all the experiments. Figure plotting was conducted with the aid of software GraphPad Prism 5 (GraphPad Software Inc., San Diego, CA, USA).

## Results

### 

#### CPTT method for an efficient culture of ASM cells

**Figure 2.**
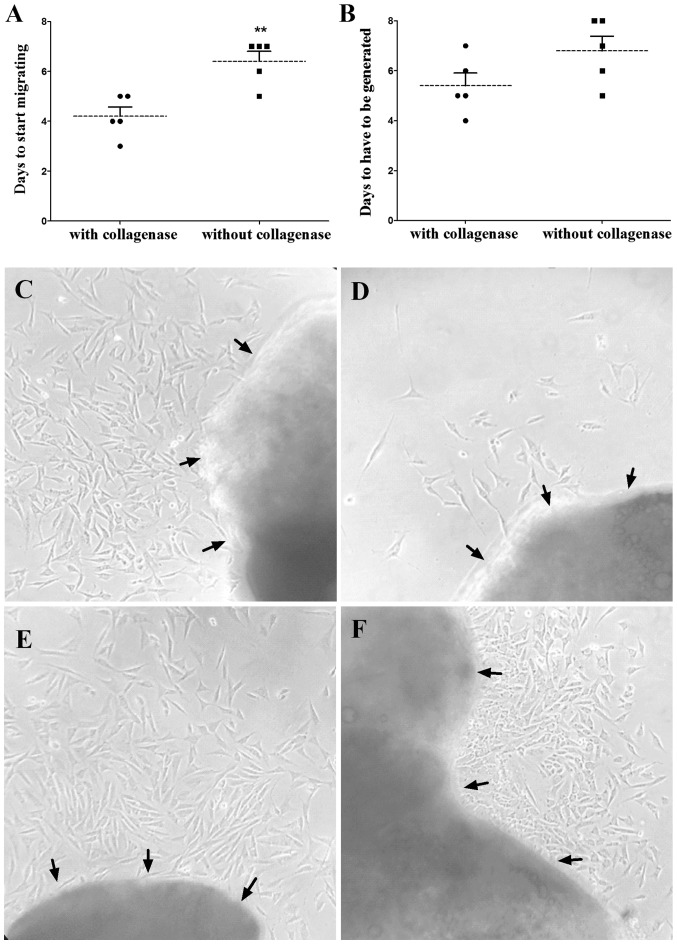
Comparison between the two different methods of culturing guinea pig airway smooth muscle (ASM) cells with or without collagenase to pretreat the ASM pieces. Number of days for the cells to (A) begin migrating out of the ASM pieces and the (B) cells having to generate due to their thick density. Data from five independent experiments were used and expressed as mean ± standard error of the mean. **P<0.01 by independent samples t-test with SPSS 16.0. The status of the cells on day 6 in the (C) with or (D) without collagenase groups, and that on days 2–4 of the ASM cells migrating from the second-hand pieces treated with the two methods is shown in (E) and (F), respectively (magnification, x100). Arrows in (C-F) indicate the ASM pieces.

As is shown in Fig. 2, the number of days for the ASM cells to start migrating out of the ‘tissue blocks’ (Fig. 2A) were significantly different between the groups treated with the two methods. The number of days for the culture to generate, as it comprised too many cells (Fig. 2B), appeared to be different between the two groups. The average time was 4.2 days for the cells to start growing out in the culture pretreated with collagenase, which was less than that (6.4 days) in the culture without pretreatment with the enzyme. The time for the dense cells to be initially passed was 5.4 days in the collagenase group and 6.8 days in the collagenase-free group, which also showed a high efficacy with the CPTT method. The status of the cells in the two groups on day 6 after the guinea pig tracheal smooth muscle strip was planted is shown in Fig. 2C and D. The ASM cells in Fig. 2C were evidently thick, with a density ~80%, while those in Fig. 2D were just beginning to migrate out of the pieces at the same time. In addition, the status of the ASM cells migrating from the second-hand pieces treated with the CPTT method was better than that of the traditional method (Fig. 2E and F).

#### Morphology and viability of ASM cells

**Figure 3.**
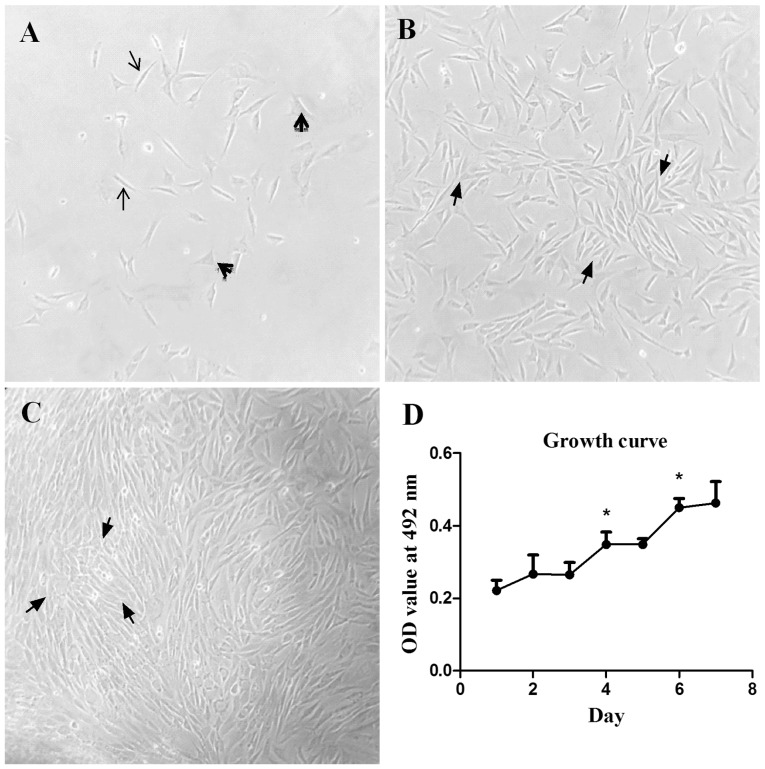
(A-C) Morphology and viability of the third generation of guinea pig airway smooth muscle cells. (D) The growth curve recording cell growth condition over time. Data are expressed as mean ± standard error of the mean (n=8). Cells from days 4 to 8 proliferate fast and the density of the cells on days 4 or 6 was significantly increased compared with that on day 1 (*P<0.05). Images of the cells, i.e. (A-C), were respectively taken under an optical microscope (magnification, x100) on days 2, 4 and 6 after generation. The arrows in (A) indicate the cells in irregular triangle form and the arrowheads in fusiform, those in (B) expressed the cells in a good state, and those in (C) are the cells on ~day 6 that aligned so closely that their morphology became abnormal.

Cells migrating from the ASM pieces treated with collagenase began to attach to the surface of the culture flask 6 h after they were generated and spread out gradually in the following 2 days. Their morphology was expressed in fusiform shown with arrowheads or an irregular triangle shown with arrows in Fig. 3A. The density of the cells became significantly thick on day 4 and they were in a good state (Fig. 3B). The typical peak-valley pattern of ASM cells was observed under an optical microscope ~day 6. However, this was at the same time that the cells aligned so closely that their morphology looked abnormal in certain local areas, which is shown with arrows in Fig. 3C.

Cell viability was determined with the MTT assay at days 1 to 7, respectively, after they were generated. As was observed in the growth curve of Fig. 3D, OD values increased significantly on days 4 and 6 (P<0.05), which indicated that the cells proliferated significantly.

#### Identification of ASM cells

**Figure 4.**
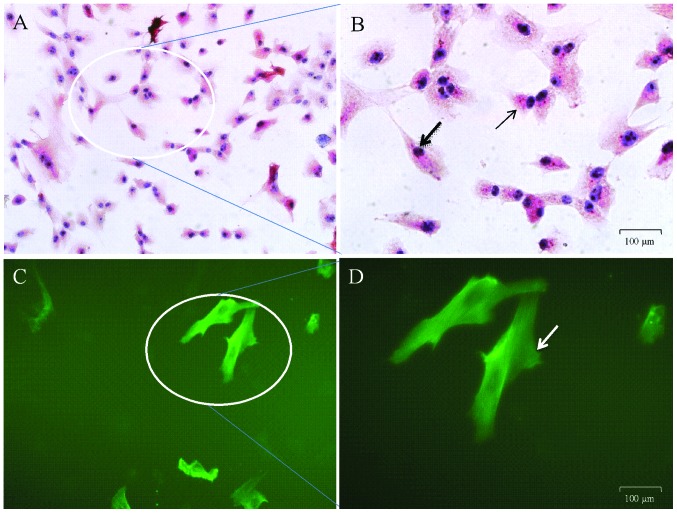
Data of (A and B) immunocytochemical identification of the cells preloaded with α-smooth muscle actin and (C and D) immunofluorescent identification of the cells preloaded with fluorescein isothiocyanate. The images in (A) and (B) were obtained under an inverted system microscope (magnification, x200 and x400, respectively) and those in (C) and (D) under an inverted fluorescent microscope (magnification, x200 and x400, respectively) in the condition of absorption peak at 492 nm and emission peak at 520 nm. The magnified cells in (B) are various shapes, including irregular triangle and fusiform, are illustrated with a thin arrow and common arrow, respectively. The arrow in (D) also indicates airway smooth muscle cells in fusiform identified with immunofluorescent staining.

Guinea pig ASM cells were identified with immunocytochemistry and immunofluorescent staining subsequently to being loaded with the specific α-SMA antibody. The results of immunocytochemistry were as stated in Fig. 4A and B. The magnified cells in Fig. 4 were in various shapes, including the irregular triangle form indicated with thin arrows and fusiform with common arrows. Green fluorescence could be observed at 492 nm under an inverted fluorescent microscope once the cells were loaded with FITC for identification with immunofluorescent staining, as expressed in Fig. 4C and D. The arrow in Fig. 4D indicates ASM cells in the fusiform. It was found that >95% of the cells were ASM cells in several randomly chosen perspectives.

#### Mab suppresses the increase of intracellular Ca^2+^ induced by Ach

##### Fluorescent intensity of Ca^2+^ determined with the Varioskan Flash

**Figure 5.**
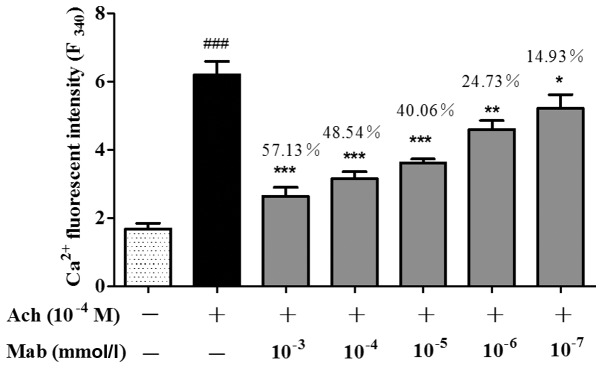
Suppression of mabuterol hydrochloride (Mab) (10^−3^, 10^−4^, 10^−5^, 10^−6^ and 10^−7^ mmol/l) on the increased level of intracellular Ca^2+^ induced by acetylcholine (Ach) (10^−4^ M) in a concentration-dependent manner. Data are expressed as mean ± standard error of the mean obtained from three independent experiments. *P<0.05, **P<0.01 and ***P<0.001 compared to the group treated with Ach, and ^###^P<0.001 compared to the control by analysis of variance followed by least significant difference with SPSS 16.0. The percentage on the top of each column indicates the inhibitory rate of intracellular Ca^2+^.

As shown in Fig. 5, Ach (10^−4^ M) significantly increases Ca^2+^ fluorescent intensity when it was determined with the multimode microplate reader. Mab (10^−3^, 10^−4^, 10^−5^, 10^−6^ and 10^−7^ mmol/l) significantly suppressed this increase in a concentration-dependent manner. The inhibitory rates of intracellular Ca^2+^ at different concentrations of Mab, from low to high, were 14.93, 24.73, 40.06, 48.54 and 57.13%, respectively (Fig. 5).

#### Fluorescent intensity of Ca^2+^ observed under the inverted system microscope

**Figure 6.**
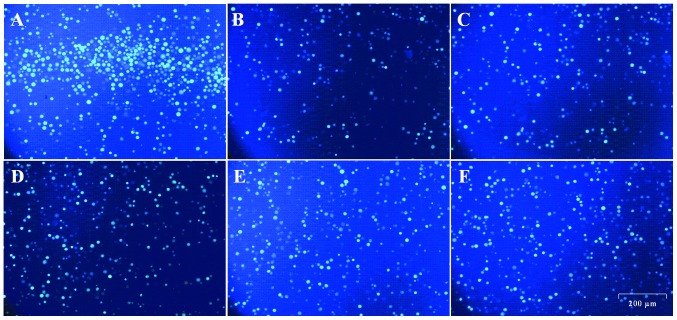
Representative image of Ca^2+^ fluorescence obtained from the inverted system microscope (magnification, x200). The image in (A) comes from the sample treated with acetylcholine (Ach) (10^−4^ mmol/l) alone and (B-F) are from the samples pre-incubated with 10^−3^, 10^−4^, 10^−5^, 10^−6^ and 10^−7^ mmol/l of mabuterol hydrochloride, respectively, and subsequently with Ach (10^−4^ mmol/l). The scale bar represents 200 µm.

Representative images of Ca^2+^ fluorescence obtained from the inverted fluorescent microscope are indicated in Fig. 6. More fluorescent spots and higher fluorescent intensity can be observed in the sample treated with Ach (Fig. 6A) compared with those in the samples pre-incubated with 10^−3^, 10^−4^, 10^−5^, 10^−6^ and 10^−7^ mmol/l of Mab and subsequently with Ach (Fig. 6B–F). The fluorescent intensity of the sample treated with the highest concentration of Mab, 10^−3^ mmol/l (Fig. 6B), was the least among all the Mab-treated samples (Fig. 6B–F).

#### Intracellular Ca^2+^ levels determined with immunocytometry systems

**Figure 7.**
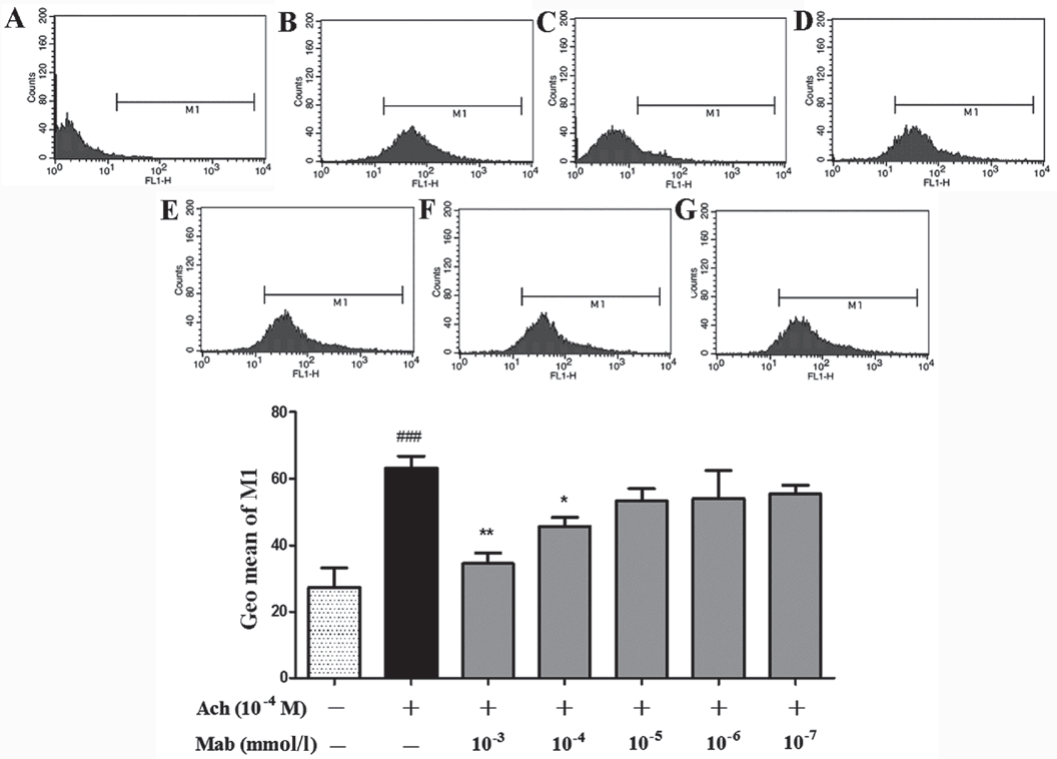
Geometric mean (Geo Mean) of range M1 in the column figure, calculated based on the diagrams of the immunocytometry systems. The cells loaded with Fluo-3/AM were respectively treated with 10^−7^, 10^−6^, 10^−5^, 10^−4^ and 10^−3^ mmol/l of mabuterol hydrochloride (Mab), and subsequently, their range of M1 in the flow cytometry diagram was decreased in a concentration-dependent manner as shown in (G), (F), (E), (D) and (C). The diagram in (B) illustrates the cells treated with 10^−4^ M acetylcholine (Ach) alone and (A) without treatment. Data are expressed as mean ± standard error of the mean obtained from three independent experiments. *P<0.05 and **P<0.01 compared to the group treated with Ach, and ^###^P<0.001 compared to the control by analysis of variance followed by least significant difference using SPSS 16.0.

The geometric mean (Geo Mean) of the M1 range in the diagram of flow cytometry (Fig. 7) was analyzed to determine Ca^2+^ fluorescent intensity. Ach (10^−4^ M) significantly increases intracellular Ca^2+^ compared with the control and Mab at the concentration of 10^−3^ mmol/l, and 10^−4^ mmol/l significantly suppresses the elevation of Ca^2+^ fluorescent intensity induced by Ach. When assessing the peak in Fig. 7C, the peak of the cells loaded with Fluo-3/AM following treatment with the highest concentration of Mab, 10^−3^ mmol/l, shifts significantly to the left compared with that in Fig. 7B (treated with Ach 10^−4^ M alone). Geo Mean in the flow cytometry diagram of the cells treated with Mab decreases in a concentration-dependent manner, as shown from Fig. 7G to Fig. 7C.

## Discussion

ASM cells are involved in the pathophysiology of numerous airway diseases, such as airway remodeling and intracellular calcium overload (15). The cell has become of interest in the study of the mechanisms of bronchial asthma and chronic obstructive pulmonary disease.

Currently, two methods, i.e. with and without enzymes to digest ASM pieces, are commonly used to prepare primary ASM cells. It is known that the former may obtain the cells in a short period of time (16). However, it may lead to a less successful rate of the cell preparation, as the ASM cells are extremely susceptible to injury from physical or chemical factors. The reasons include that it is difficult to control the exact quantity of enzymes and digestion time in addition to the vulnerability of the cells. By contrast, not using enzymes is relatively simple to handle and the step is mild for the cells, but it is difficult for the cells to migrate out of the tissue block due to its untreated toughness, and therefore, the culture time is extended (17). An improved method without CPTT was discussed in a previous study (11). In this study, the culture method with CPTT was tested and various periods of time, i.e. 5, 10, 20, 30 and 40 min, were assessed respectively to examine which was the optimum time for enzyme digestion; 20 min was the best treatment time. The time for the ASM cells to migrate out of the tissue block was not fast compared with that of the method without CPTT, as the time for digestion was not long enough. By contrast, tissue blocks may become cotton- and wool-like and too many cells were subjected to the enzymatic digestion if the time was too long. Tissues digested after an appropriate duration in the present study became softer and less tough, which caused the easier and earlier migration of the cells from the tissue blocks. Additionally, tissue blocks treated with collagenase can be repeatedly used ≤3 times. Through trial and error experimental conditions, the cell culture method established in the study is rapid, simple, efficient and reproducible, which may provide a good platform for *in vitro* studies in this research area.

The function of ASM cells is clearly regulated by various signaling molecules. Activation of enzymes, protein phosphorylation and release of calcium pools are all involved in the transduction of the signaling molecules. Among them, Ca^2+^ may play a central role and free Ca^2+^ in the cytosol of ASM cells acts as a crucial secondary messenger in numerous biological processes, such as contraction, proliferation, gene transcription and secretion of signal mediators (4). Ach-induced Ca^2+^ transients and oscillations in ASM cells have been previously studied and reported (18,19). The present study identified that Mab significantly suppressed the increased level of intracellular Ca^2+^ induced by Ach.

The drug is a selective long-acting β_2_-receptor agonist to be clinically used for asthma treatment (20). In the present study, to evaluate the mechanism of the antiasthmatic effect of Mab, several methods of Ca^2+^ measurement were used, including quantitative and qualitative analysis. Two calcium fluorescent probes (Fura-2/AM and Fluo-3/AM) and three detection methods were applied to determine Ca^2+^ fluorescent intensity. Images of Ca^2+^ fluorescence in Fig. 6 illustrated the suppressive effect of Mab on the increased Ca^2+^, which provided the information regarding the drug's action though the measurement with the inverted fluorescent microscope, as a method of qualitative analysis. Additionally, Ca^2+^ fluorescent intensity was quantified through single-wavelength detection with a multimode microplate reader. As is illustrated in Fig. 5, Mab concentration-dependently inhibits intracellular Ca^2+^, which is clearly exhibited by the calcium inhibition rates. In addition, the high concentration of Mab (10^−3^ and 10^−4^ mmol/l) significantly suppressed the elevation of Ca^2+^ fluorescent intensity induced by Ach, which was obtained from the Geo Mean of range M1 in the flow cytometry diagram (Fig. 7). A total of 10,000 cells in each group were automatically captured and analyzed with the equipment so as to compare the data of the groups. Similar conclusions arose with these detective methods. Mab significantly inhibits the Ca^2+^ increase induced by Ach in guinea pig ASM cells.

**Figure 8.**
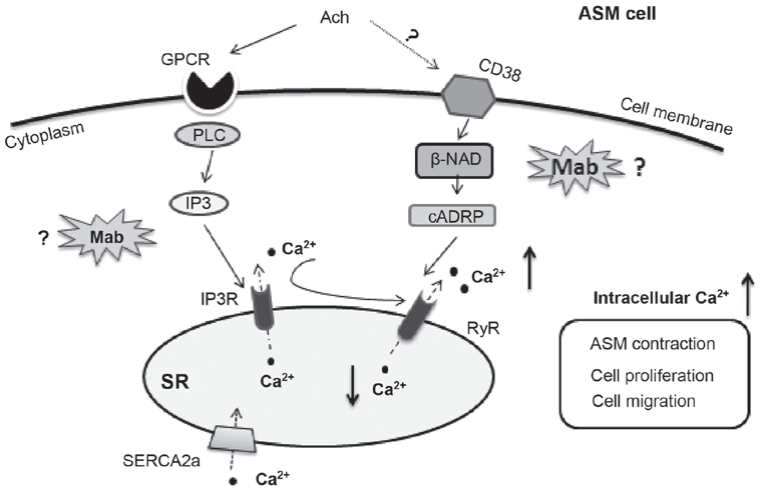
Signaling pathways of Ca^2+^ mobilization in an airway smooth muscle (ASM) cell involving the inositoltrisphosphate receptor (IP3R) and ryanodine receptor (RyR), and the potential targets of mabuterol hydrochloride (Mab) that intervene in the increased level of intracellular Ca^2+^ induced with Ach. Binding with a G-protein coupled receptor, Ach activates phospholipase C (PLC) to generate IP3, which encourages the clusters of IP3R on SR to release Ca^2+^. This process may stimulate the adjacent RyR to increase Ca^2+^. RyR may also be activated or potentiated by cADP ribose (cADPR). It may be sequestered by the superficial sarcoplasmic reticulum (SR) through sarcoendoplasmic Ca^2+^ ATPase 2a (SERCA2a), although much of the calcium is released from stores and enters the cytoplasm. The increased level of intracellular Ca^2+^ leads to the contraction, proliferation and migration of the ASM.

Ach binds G-protein coupled receptors on the membrane of ASM cells to activate phospholipase C, and subsequently, inositoltrisphosphate (IP3) is produced under its catalysis (21). In addition, Ach can stimulate cluster of differentiation 38 to generate cADP-Ribose (cADPR). Ca^2+^ is released from SR into the cytoplasm subsequent to IP3 stimulating the clusters of IP3 receptors (IP3Rs) on the membrane of SR and/or cADPR stimulating the ryanodine receptor (RyR) (Fig. 8) (22). There is a possibility of RyR increasing again via the mechanism of calcium-induced calcium release following the activation of IP3R when the level of intracellular Ca^2+^ is high enough, which leads to the evacuation of the SR store and the accumulation of cytoplasmic Ca^2+^ (23). Intracellular Ca^2+^ accumulation eventually results in ASM contraction, proliferation and migration. Our previous study identified that the mechanism of tradinterol suppression on the elevation of intracellular Ca^2+^ may be involved in the IP3R pathway (11). As was suggested in previous studies, tradinterol is a new type of long-acting β_2_-agonist (24,25). In the present study, Mab was proved to significantly inhibit the calcium increase induced by Ach in guinea pig ASM cells. Further investigation of whether the suppressive activity of the drug on the calcium is the result of the interaction between IP3R and RyR signaling pathways (Fig. 8) is required.

In conclusion, the renewed method of ASM cell culture has successfully been proved. Additionally, it is clearly shown that Mab significantly suppresses the increased level of intracellular Ca^2+^ induced by Ach through three measurement methods with a specific fluorescent probe in the ASM cells. Due to the mechanism of calcium increase induced with Ach and the suppressive effect of Mab on the increased level of intracellular Ca^2+^, more studies should be performed to clarify the mechanism of the suppression in detail, in which RyR and/or the IP3R signaling pathway may provide innovative ideas with further research.

## References

[1] James A, Mauad T, Abramson M, Green F (2012). Airway smooth muscle hypertrophy and hyperplasia in asthma. Am J Respir Crit Care Med.

[2] Siddiqui S, Redhu NS, Ojo OO, Liu B, Irechukwu N, Billington C, Janssen L, Moir LM (2013). Emerging airway smooth muscle targets to treat asthma. Pulm Pharmacol Ther.

[3] Noble PB, Pascoe CD, Lan B, Ito S, Kistemaker LE, Tatler AL, Pera T, Brook BS, Gosens R, West AR (2014). Airway smooth muscle in asthma: Linking contraction and mechanotransduction to disease pathogenesis and remodelling. Pulm Pharmacol Ther.

[4] Koopmans T, Anaparti V, CastroPiedras I, Yarova P, Irechukwu N, Nelson C, PerezZoghbi J, Tan X, Ward JP, Wright DB (2014). Ca^2^ handling and sensitivity in airway smooth muscle: Emerging concepts for mechanistic understanding and therapeutic targeting. Pulm Pharmacol Ther.

[5] Boese M, Busse R, Mülsch A, Schini-Kerth V (1996). Effect of cyclic GMP-dependent vasodilators on the expression of inducible nitric oxide synthase in vascular smooth muscle cells: Role of cyclic AMP. Br J Pharmacol.

[6] Dimitropoulou C, White RE, Ownby DR, Catravas JD (2005). Estrogen reduces carbachol-induced constriction of asthmatic airways by stimulating large-conductance voltage and calcium-dependent potassium channels. Am J Respir Cell Mol Biol.

[7] Wang IY, Bai Y, Sanderson MJ, Sneyd J (2010). A mathematical analysis of agonist- and KCl-induced Ca(2+) oscillations in mouse airway smooth muscle cells. Biophys J.

[8] Yamamoto H, Nagata M, Tabe K, Suzuki S, Maruo H, Sakamoto Y, Yamamoto K, Dohi Y (1990). The inhibitory effect of long-acting beta-adrenergic agonists, mabuterol, clenbuterol and fenoterol on ‘morning dipping’ in patients with asthma. Arerugi.

[9] Osada E, Murai T, Ishizaka Y, Sanai K (1984). Pharmacological studies of mabuterol, a new selective beta 2-stimulant. II: Effects on the cardiovascular system and smooth muscle organs. Arzneimittelforschung.

[10] Akahane K, Furukawa Y, Ogiwara Y, Haniuda M, Chiba S (1989). Beta-adrenoceptor blocking effects of a selective beta 2-agonist, mabuterol, on the isolated, blood-perfused right atrium of the dog. Br J Pharmacol.

[11] Liu J, Zhang Y, Li Q, Zhuang Q, Zhu X, Pan L, Cheng M (2014). An improved method for guinea pig airway smooth muscle cell culture and the effect of SPFF on intracellular calcium. Mol Med Rep.

[12] Mosmann T (1983). Rapid colorimetric assay for cellular growth and survival: Application to proliferation and cytotoxicity assays. J Immunol Methods.

[13] Orlandi A, Calzetta L, Doldo E, Tarquini C, Matera MG, Passeri D (2015). Brain natriuretic peptide modulates calcium homeostasis and epidermal growth factor receptor gene signalling in asthmatic airways smooth muscle cells. Pulm Pharmacol Ther.

[14] Liu B, Yang J, Wen Q, Li Y (2008). Isoliquiritigenin, a flavonoid from licorice, relaxes guinea-pig tracheal smooth muscle in vitro and in vivo: Role of cGMP/PKG pathway. Eur J Pharmacol.

[15] Pelaia G, Renda T, Gallelli L, Vatrella A, Busceti MT, Agati S, Caputi M, Cazzola M, Maselli R, Marsico SA (2008). Molecular mechanisms underlying airway smooth muscle contraction and proliferation: Implications for asthma. Respir Med.

[16] Yamakage M, Hirshman CA, Croxton TL (1995). Volatile anesthetics inhibit voltage-dependent Ca^2^ channels in porcine tracheal smooth muscle cells. Am J Physiol.

[17] Wu BN, Lin RJ, Lo YC, Shen KP, Wang CC, Lin YT, Chen IJ (2004). KMUP-1, a xanthine derivative, induces relaxation of guinea-pig isolated trachea: The role of the epithelium, cyclic nucleotides and K channels. Br J Pharmacol.

[18] Bergner A, Sanderson MJ (2002). Acetylcholine-induced calcium signaling and contraction of airway smooth muscle cells in lung slices. J Gen Physiol.

[19] Perez JF, Sanderson MJ (2005). The frequency of calcium oscillations induced by 5-HT, ACH and KCl determine the contraction of smooth muscle cells of intrapulmonary bronchioles. J Gen Physiol.

[20] Kawakami Y (1984). First clinical studies on mabuterol. A summarizing report. Arzneimittelforschung.

[21] Gosens R, Zaagsma J, Grootte Bromhaar M, Nelemans A, Meurs H (2004). Acetylcholine: A novel regulator of airway smooth muscle remodelling?. Eur J Pharmacol.

[22] Jude JA, Wylam ME, Walseth TF, Kannan MS (2008). Calcium signaling in airway smooth muscle. Proc Am Thorac Soc.

[23] Mahn K, Ojo OO, Chadwick G, Aaronson PI, Ward JP, Lee TH (2010). Ca(2+) homeostasis and structural and functional remodelling of airway smooth muscle in asthma. Thorax.

[24] Gan LL, Wang MW, Cheng MS, Pan L (2003). Trachea relaxing effects and beta2-selectivity of SPFF, a newly developed bronchodilating agent, in guinea pigs and rabbits. Biol Pharm Bull.

[25] Hao Z, Zhang Y, Pan L, Su X, Cheng M, Wang M, Zhao H, Wu Y (2008). Comparison of enantiomers of SPFF, a novel beta2-Adrenoceptor agonist, in bronchodilating effect in guinea pigs. Biol Pharm Bull.

